# Rational Combination of Parvovirus H1 With CTLA-4 and PD-1 Checkpoint Inhibitors Dampens the Tumor Induced Immune Silencing

**DOI:** 10.3389/fonc.2019.00425

**Published:** 2019-05-28

**Authors:** Katrin Goepfert, Christiane Dinsart, Jean Rommelaere, Friedrich Foerster, Markus Moehler

**Affiliations:** ^1^Department of Medicine I, University Medical Center of the Johannes Gutenberg University Mainz, Mainz, Germany; ^2^Infection, Inflammation and Cancer Program, Tumor Virology Division (F010), German Cancer Research Center (DKFZ), Heidelberg, Germany

**Keywords:** melanoma, immune cells, H-1PV, nivolumab, ipilimumab, immunotherapy

## Abstract

The recent therapeutic success of immune checkpoint inhibitors in the treatment of advanced melanoma highlights the potential of cancer immunotherapy. Oncolytic virus-based therapies may further improve the outcome of these cancer patients. A human *ex vivo* melanoma model was used to investigate the oncolytic parvovirus H-1 (H-1PV) in combination with ipilimumab and/or nivolumab. The effect of this combination on activation of human T lymphocytes was demonstrated. Expression of CTLA-4, PD-1, and PD-L1 immune checkpoint proteins was upregulated in H-1PV-infected melanoma cells. Nevertheless, maturation of antigen presenting cells such as dendritic cells was triggered by H-1PV infected melanoma cells. Combining H-1PV with checkpoint inhibitors, ipilimumab enhanced TNFα release during maturation of dendritic cells; nivolumab increased the amount of IFNγ release. H-1PV mediated reduction of regulatory T cell activity was demonstrated by lower TGF-ß levels. The combination of ipilimumab and nivolumab resulted in a further decline of TGF-ß levels. Similar results were obtained regarding the activation of cytotoxic T cells. H-1PV infection alone and in combination with both checkpoint inhibitors caused strong activation of CTLs, which was reflected by an increased number of CD8^+^GranB^+^ cells and increased release of granzyme B, IFNγ, and TNFα. Our data support the concept of a treatment benefit from combining oncolytic H-1PV with the checkpoint inhibitors ipilimumab and nivolumab, with nivolumab inducing stronger effects on cytotoxic T cells, and ipilimumab strengthening T lymphocyte activity.

## Introduction

In recent years, major advances have been achieved in the treatment of advanced melanoma based on a better understanding of the interaction between melanoma cells and the cells of the human immune system (1). Consequently immune-based therapies including ipilimumab and nivolumab or oncolytic virus therapies such as Talimogene laherparepvec (T-VEC) have been developed and approved for the treatment of melanoma patients leading to an improvement in response rates and progression-free survival (2–4).

Oncolytic virotherapy is an emerging therapeutic modality and offers the opportunity to treat cancer patients with tumors resistant to standard therapies. Autonomous rat parvovirus H-1 (H-1PV), a small nuclear-replicating DNA virus, has been shown to induce cell lysis in human malignant cells including colon carcinoma, melanoma and pancreatic adenocarcinoma leaving healthy cells unaffected (oncotropism) (5–8). H-1PV inoculation generates immunogenic tumor cell lysates which have been shown to induce the maturation of dendritic cells (DCs), the release of pro-inflammatory cytokines, tumor associated antigen cross presentation, and T cell stimulation in human melanoma and glioma cells (6, 9, 10). Clinical trials in patients with glioblastoma have demonstrated that H-1PV suppresses the activity of regulatory T cells (Treg) and promotes immune cell activation (CD8^+^- and CD4^+^- T lymphocytes as well as tumor associated macrophages) as indicated by increasing serum levels of perforin, granzyme B, IFNγ, and IL-2 (11–14). Although, synergistic cytotoxic effects of H-1PV combined with gemcitabine in pancreatic cancer have been demonstrated, little is known about combinations of H-1PV with other cytotoxic drugs or checkpoint inhibitors (8, 15).

The immune checkpoint inhibitors ipilimumab and nivolumab have been approved by the US Food and Drug Administration (FDA) and the European Medicine Agency (EMA) for the treatment of metastatic or unresectable advanced melanoma. Ipilimumab is a monoclonal antibody that blocks cytotoxic T lymphocyte associated protein-4 (CTLA-4). CTLA-4 is constitutively expressed by regulatory T cells (Tregs) and activated T helper cells. Furthermore, CTLA-4 expression on freshly isolated monocytes and matured dendritic cells was accompanied by an increased IL-10 release (16). Interaction of CTLA-4 with its ligands inhibits T cell activation by preventing their proliferation, cell cycle progression, and production of pro-inflammatory cytokines such as IL-2 (17). In addition several studies have shown that CTLA-4 expression inhibits anti-tumor immune responses. CTLA-4 is also expressed on human tumor cell lines including breast carcinoma, osteosarcoma, colon carcinoma and melanoma (18, 19). Furthermore, IFNγ (TME) activates the expression of CTLA-4 in the tumor microenvironment (20). This may explain how some tumors escape the human immune response.

Nivolumab engages with the programmed cell death protein 1 (PD-1) on the surface of melanoma cells and immune cells including natural killer cells and myeloid suppressor cells (21). Similar to CTLA-4, PD-1 serves as a negative regulator of T cell activation and is upregulated after activation of T cells. Binding of PD-1 with its ligand, programmed death ligand 1 (PD-L1), in the TME, inhibits the activity of cytotoxic T cells (CTL), induces apoptosis of tumor infiltrating T cells and moves differentiation toward Tregs (22, 23).

Tregs play an important role in preventing anti-tumor immune responses (24). Enrichment of Tregs has been shown in the TME of human melanomas and in the peripheral blood of cancer patients (25). Different mechanisms have been described for Treg-mediated immune suppression which were shown to be driven by the release of transforming growth factor beta (TGF-ß) and also through direct interaction with T helper cells (26). Tregs express high levels of CTLA-4 and PD-1 on their surface. Therefore, the immune checkpoint inhibitors ipilimumab and nivolumab have a dual mode of action, directly targeting tumor cells expressing CTLA-4, PD-1, and PD-L1, and also by inhibiting the Treg response to tumors.

A combination of oncolytic virotherapy and checkpoint inhibition may overcome the tumor-induced immunosuppressive milieu through different mechanisms. For example, direct infection of melanoma cells by H-1PV leads to tumor lysis and simultaneous stimulation of an immune response (9, 27, 28). Combination with ipilimumab and nivolumab may further enhance immune stimulation by preventing immune silencing effects including Treg activation or CTLA-4 and PD-1 expression on tumor and immune cells. In this study, we now tested this concept by combining H-1PV infection with ipilimumab and nivolumab in our human *ex vivo* melanoma model with lymphocytes of the same patient (29, 30). The first step was to determine the effect on the maturation of DCs, which have been selected out of corresponding HLA-A2 restricted buffy coats and secondly to test the effect on the patient specific T lymphocytes, in particular HLA-A2 restricted Tregs from the same buffy coats.

To our knowledge, this is the first investigation of a combination of oncolytic parvovirus with immune checkpoint inhibitors. Our intention was to provide a preclinical rationale for the investigation of new therapeutic combinations in clinical studies.

## Materials and Methods

### Human Melanoma Cell Lines and H-1PV Infection

The melanoma cell lines Sk29Mel-1 and Mz7Mel (both a gift from Prof. T. Woelfel's group) were cultured in RPMI 1640 medium (Invitrogen, Carlsbad, US) supplemented with 10% fetal calf serum (Invitrogen) and 1% penicillin/streptomycin (Invitrogen) at 37% in 5% CO_2_ atmosphere. Sk29Mel had been isolated in 1975 from a recurrent melanoma and expressed melanoma specific antigens such as Melan-A and MAGE-3 (31–33). Similar melanoma specific antigens were found on Mz7Mel which had been isolated from a metastatic site in the spleen in 1988 (33). Both cell lines express HLA-A2. Infection of Sk29Mel-1 and Mz7Mel with H-1PV was performed as described previously (6, 28). H-1PV was provided by Prof. J. Rommelaere (German Cancer Research Center, Heidelberg, Germany). Briefly, the cell culture medium was removed, and the melanoma cells were incubated with H-1PV at a multiplicity of infection (MOI) of 15 plaque forming units (PFU) per cell. After 1 h, the culture medium was restored to the original volume and incubated for another 5 days. Before co-culturing infected cells with immune cells, media was completely removed and replaced.

### DC Differentiation and Maturation

Peripheral blood mononuclear cells (PBMCs) were generated from human buffy coats (delivered by the Department of Transfusion Medicine, University Medical Centre Mainz, Mainz, Germany) from healthy donors by Ficoll-Paque gradient centrifugation. Subsequently, monocytes were isolated by adherence (34). To induce the differentiation of the purified monocytes into immature (i)DCs, monocytes were cultured in X-VIVO (Biozym, Hessisch Oldendorf, Germany) supplemented with 1% plasma, IL-4 (Immunotools, Friesoythe, Germany) and granulocyte macrophage colony stimulating factor (Sandoz, Nürnberg, Germany) as described previously (6). Maturation of DCs was induced by addition of a cytokine cocktail containing 10 ng/ml TNFα (Biomol, Hamburg, Germany), 1,000 IU/ml IL-6 (Miltenyi Biotec, Bergisch Gladbach, Germany), 10 ng/ml IL-1ß (Miltenyi Biotec) and 1 μg/ml PGE2 (Merck KGaA, Darmstadt, Germany) for 3 days.

### Co-culture of iDCs With Melanoma Cell Lines

Isolated iDCs were co-cultured with melanoma cells (Sk29Mel-1 and Mz7Mel) in a ratio of 1:5 (melanoma cells:iDC) (6). Ipilimumab was used at a final concentration of 19.4 μg/ml and nivolumab at 75.3 μg/ml. Both checkpoint inhibitors were supplied by Bristol-Myers Squibb GmbH & Co KG (Munich, Germany). After 3 days, the maturation status was determined by extracellular staining and measurement of CD80, CD83, and CD86 (Becton Dickinson; Heidelberg, Germany) via fluorescence-activated cell sorting (FACS) and by measurement of the interleukin-6 (IL-6), interferon gamma (IFNγ), and tumor necrosis factor alpha (TNFα) levels in the supernatant via an enzyme linked immunosorbent assay (ELISA; Thermo Fisher Scientific, Darmstadt, Germany).

### *In vitro* T-Lymphocyte Co-culture Experiments

Human Tregs were isolated from the non-adherent fraction of PBMCs in two steps. We used a separation protocol which was established by Prof Jonuleit's group (35). First CD25+ cells were separated with CD25 magnetic Beads (MiltenyiBiotec, Bergisch Gladbach, Germany) and in a second step Tregs were purified by negative separation with CD8, CD14, CD19 dynal beads (Becton Dickinson, Heidelberg, Germany). Next, Tregs were co-cultured with melanoma cell stimulated iDCs in a ratio of 1:5 (DCs:Tregs) 24 h after co-culturing DCs with melanoma cells. To test the effect of nivolumab and ipilimumab on Tregs, both checkpoint inhibitors were added in the course of the mixed lymphocyte reaction and not during co-culture of melanoma cells with DCs. Activation of Tregs was verified by expression of the activation marker Granzyme B (via FACS Becton Dickinson, Heidelberg, Germany) on the surface of CD4^+^FoxP3^+^ gated cells and release of TGF-ß and IL-10 (Thermo Fisher Scientific, Darmstadt, Germany) using ELISA after 2 days of co-culture.

The cytotoxic T cell clone IVSB (a gift from Prof. T. Woelfel) recognizes specific antigens of Sk29Mel-1 cells in association with HLA-A2 (29, 30). IVSB were co-cultured with Sk29Mel-1 which were pre-stained with 5 μM CPD eFluor 670 [(CPD) Thermo Fisher Scientific, Darmstadt, Germany] in a ratio of 1:5 and 1:1 (melanoma cell:CTL) to test cytotoxicity. After 2 days cells were harvested and stained for CD3, CD8, and propidium iodide [PI(10 μg/ml)Merck KGaA, Darmstadt, Germany]. Cytotoxicity of CTLs was determined by expression of CPD^+^ PI^+^CD3^−^CD8 cells (36).

### Flow Cytometry

FACS analysis was used for the detection of PD-1, CTLA-4, and PD-L1 (Becton Dickinson, Heidelberg, Germany) on the surface of melanoma cells in dependence of H-1PV infection. Furthermore, maturation markers such as CD80, CD83, and CD86 (Becton Dickinson, Heidelberg, Germany) were used to determine DC maturation, and Granzyme B (Becton Dickinson, Heidelberg, Germany) to represent T cell activity. FACS staining was performed as recommended by the manufacturer's data sheets. Shortly, cells were harvested and washed twice with Wash buffer (PBS+2.5% FBS). FACS antibodies were added in concentrations as recommended by the manufacturer's data sheets and incubated at room temperature. After 20 min cells were washed twice with wash buffer and measured immediately. For intracellular staining, cells were treated for 20 min with fixation/permeabilization buffer (Becton Dickinson, Heidelberg, Germany). After washing with Perm/Wash buffer (Becton Dickinson, Heidelberg, Germany) antibodies were added and incubated for 30 min at 4°C. Cells were washed twice and analyzed by FACS.

Fluorescence was measured with a minimum of 25,000 events per sample in a FACSCalibur (BD Bioscience, Heidelberg, Germany) according to the manufacturer's instructions. Data analyses were performed using Cell Quest Pro software (BD Bioscience).

### ELISA

Supernatants of co-cultured cells were collected before harvesting and stored at −80°C. Granzyme B (GranB; R&D, Wiesbaden, Germany), IFNγ, TNFα, interleukin 10 (IL-10), TGF-ß, and IL-6 assays were performed as recommended by the manufacturer (Thermo Fisher Scientific, Darmstadt, Germany). The plates were read in the spectrophotometer (ELISA Reader, Bio-Tek Instruments, Bad Friedrichshall, Germany) at 450 nm, and values of 570 nm were subtracted to diminish background noise.

### Statistics

All shown data are illustrated as mean ± standard deviation. Differences between the groups were calculated with Student's *t*-test. A distinction is made according to ^*^P ≤ 0.05; ^**^P ≤ 0.01, and ^***^P ≤ 0.001. The *p*-value of ≤ was considered statistically significant.

## Results

### H-1PV Induced Upregulation of CTLA-4, PD-1, and PD-L1 in Human Melanoma Cells

**Figure 1:**
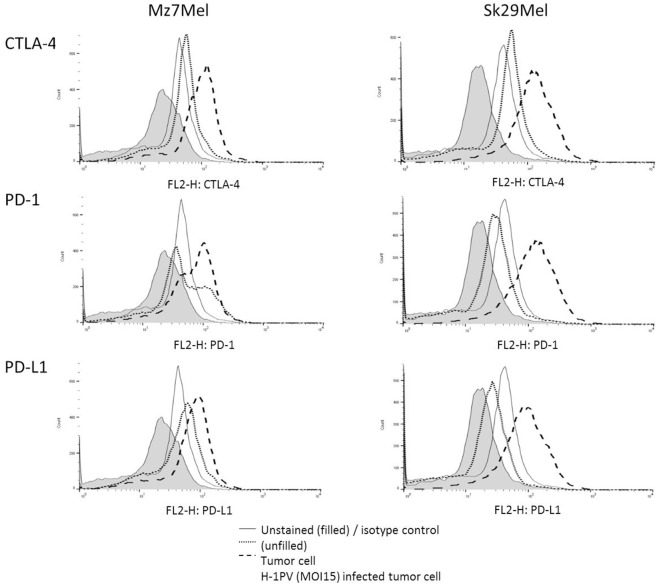
Expression of CTLA-4, PD-1, and PD-L1 on the surface of the melanoma cells Sk29Mel-1 and Mz7Mel after H-1PV infection using FACS analysis (*n* = 3). Histogram blots are illustrated in an example manner. Experiments were performed independently as triplicate. FACS, fluorescence-activated cell sorting.

Cell surface expression of immune checkpoint proteins including CTLA-4, PD-1, and PD-L1 may enable the tumor to escape the immune response or to prevent activation of T lymphocytes. To test this, we first investigated the expression levels of CTLA-4, PD-1, and PD-L1 on melanoma cells (Figure 1). Sk29Mel-1 and Mz7Mel cells demonstrated low surface levels of CTLA-4 (13% vs. 12%), PD-1 (15% vs. 11%) and PD-L1 (3% vs. 4%). Infection with H-1PV increased the expression levels of all CTL-4 (4-fold vs. 5-fold), PD-1 (3-fold vs. 5-fold), and PD-L1 (15-fold vs. 11-fold). Thus combining H-1PV treatment with ipilimumab or nivolumab to further enhance the therapeutic effect of H-1PV may be a promising approach.

### H-1PV Induced Strong DC Maturation

**Figure 2:**
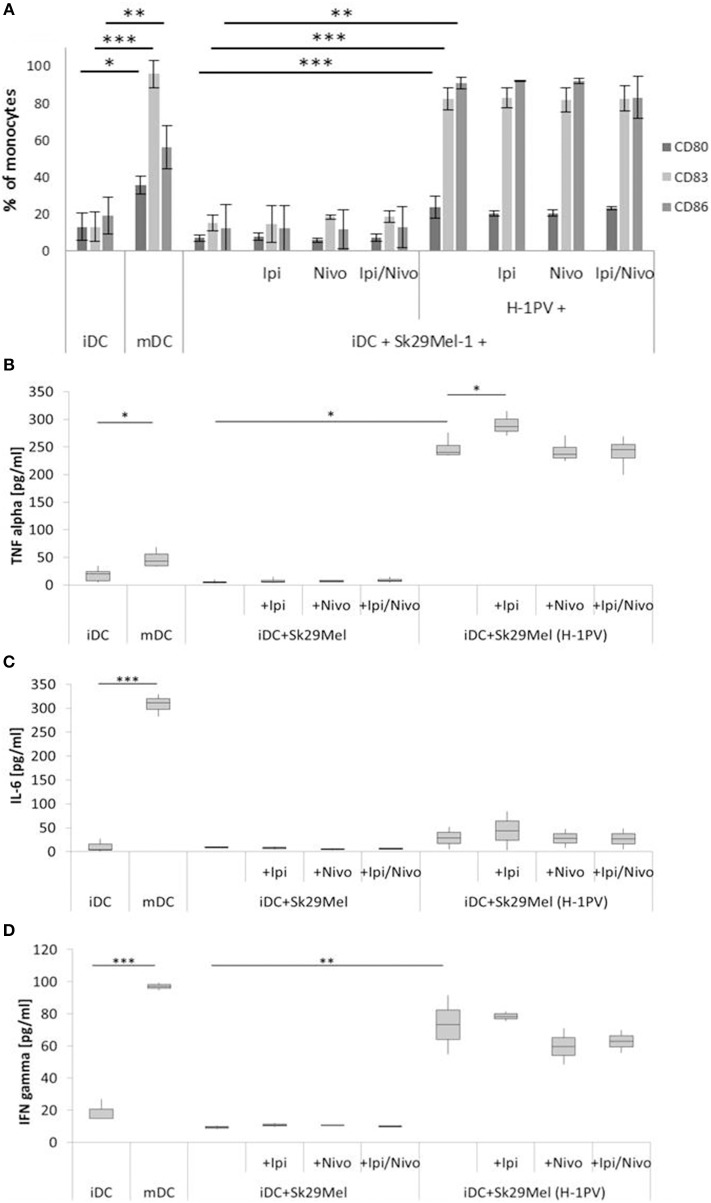
Determination of DC maturation after co-culturing of iDCs with uninfected and H-1PV infected Sk29Mel-1 cells with or without the checkpoint inhibitors ipilimumab and nivolumab for 3 days. Surface expression of the maturation markers CD80, CD83, and CD86 was determined using FACS analysis **(A)** while release of IL-6, IFNγ, and TNFα in the supernatant is determined by ELISA **(B–D)**. Experiments were performed independently as triplicate *P ≤ 0.05; **P ≤ 0.01; ***P ≤ 0.001. iDC, immatured dendritic cells; mDC, matured dendritic cells; Ipi, ipilimumab; Nivo, nivolumab; ELISA, enzyme linked immunosorbent assay; FACS, fluorescence-activated cell sorting.

**Figure 3:**
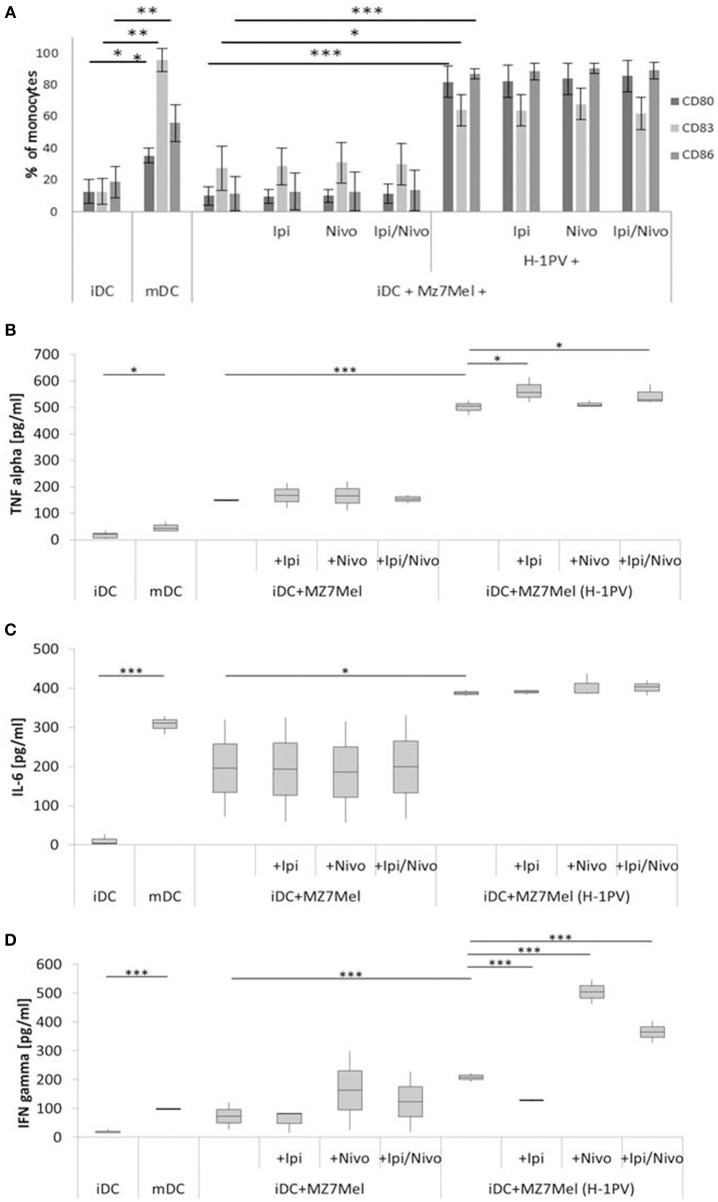
Determination of DC maturation after co-culturing of iDCs with uninfected and H-1PV infected Mz7Mel with or without the checkpoint inhibitors ipilimumab and nivolumab for 3 days. Results of three different experiments are shown. Surface expression of the maturation marker CD80, CD83 and CD86 was determined using FACS analysis **(A)** while release of IL-6, IFNγ, and TNFα in the supernatant is determined by ELISA **(B–D)**. Experiments were performed independently as triplicate *P ≤ 0.05; **P ≤ 0.01; ***P ≤ 0.001. iDC, immatured dendritic cells; mDC, matured dendritic cells; Ipi, ipilimumab; Nivo, nivolumab; ELISA, enzyme linked immunosorbent assay; FACS, fluorescence-activated cell sorting.

IDCs expressed only low levels of CD80 (14%), CD83 (49%), and CD86 (56%) and released only low levels of TNFα (18 pg/ml), IL-6 (10 pg/ml), and IFNγ (21 pg/ml). After stimulation with a cytokine cocktail containing IL-1ß, IL-6, TNFα, and PGE_2_ for 3 days, there was a strong increase in CD80 (3-fold), CD83, and CD86 (both 2-fold) as well as strengthened release of IL-6 (31-fold), TNFα (3-fold), and IFNγ (4-fold) (Figures 2,3).

As control, the release of TNFα, IL-6, and IFNγ was also determined for the untreated melanoma cells alone (data not shown). There were only small amounts of IFNγ 17 ± 4 pg/ml for Sk29Mel and 7 ± 3 pg/ml for Mz7Mel. Regarding TNFα, Sk29Mel (15 ± 7 pg/ml) released 3-fold more than Mz7Mel (5 ± 2 pg/ml). In contrast, IL-6 was much higher in the supernatant of Mz7Mel (89 ± 8 pg/ml) than in Sk29Mel (20 ± 4 pg/ml).

To examine the combination of H-1PV infection with immune checkpoint inhibitors on the activation of the human immune system, we tested the effects of ipilimumab and nivolumab on the maturation of DCs. IDCs were co-cultured with H-1PV infected or uninfected melanoma cells in combination with ipilimumab, nivolumab, or both. Sk29Mel-1 cells alone induced neither the expression of the DC maturation markers CD80, CD83, and CD86 nor the release of the pro-inflammatory cytokines IL-6, IFNγ, and TNFα (Figure 2). Conversely, H-1PV infected Sk29Mel-1 induced a 4- to 8-fold upregulation of maturation markers as well as IL-6 (3-fold), TNFα (7-fold), and IFNγ cytokines (8-fold). The addition of immune checkpoint inhibitors did not induce DC maturation or enhance H-1PV induction of DC maturation or cytokine release (Figure 2).

As shown in Figure 3, Mz7Mel cells in co-culture with DC also failed to upregulate the expression of DC maturation markers. A difference between Sk29Mel-1 and Mz7Mel cells was seen in the release of cytokines. In co-culture experiments with Mz7Mel cells, there was more TNFα (5-fold), IL-6 (22-fold), and IFNγ (8-fold) detectable. After infection with H-1PV, the level of CD80 and CD86 rose 8-fold and CD83 2-fold. There was also a greater amount of released IL-6 (2-fold), TNFα (3-fold), and IFNγ (3-fold increase).

The addition of ipilimumab and/or nivolumab did not change the proportions of CD80^+^, CD83^+^ and CD86^+^ cells. Co-culture of H-1PV infected Mz7Mel with DC induced an 11% increase in TNFα. Also, ipilimumab alone and in combination with nivolumab demonstrated higher TNFα release in co-cultures with Mz7Mel alone. In addition, strong release of IFNγ was demonstrated in combination with nivolumab as monotherapy and in combination with ipilimumab. An opposite effect, a decrease in IFNγ was shown by adding ipilimumab alone.

To conclude, both melanoma cell lines induced maturation of DCs after infection with H-1PV. The combination of checkpoint inhibitors with H-1PV infection did not increase the number of matured dendritic cells but may improve the release of cytokines such as TNFα and IFNγ. In H-1PV infected Mz7Mel cells co-cultured with iDCs, nivolumab was associated with an increased release of IFNγ, while ipilimumab was associated with an increase in TNFα levels.

### The Combination of H-1PV With Ipilimumab and Nivolumab Prevented of Immune Silencing by Treg Cells

**Figure 4:**
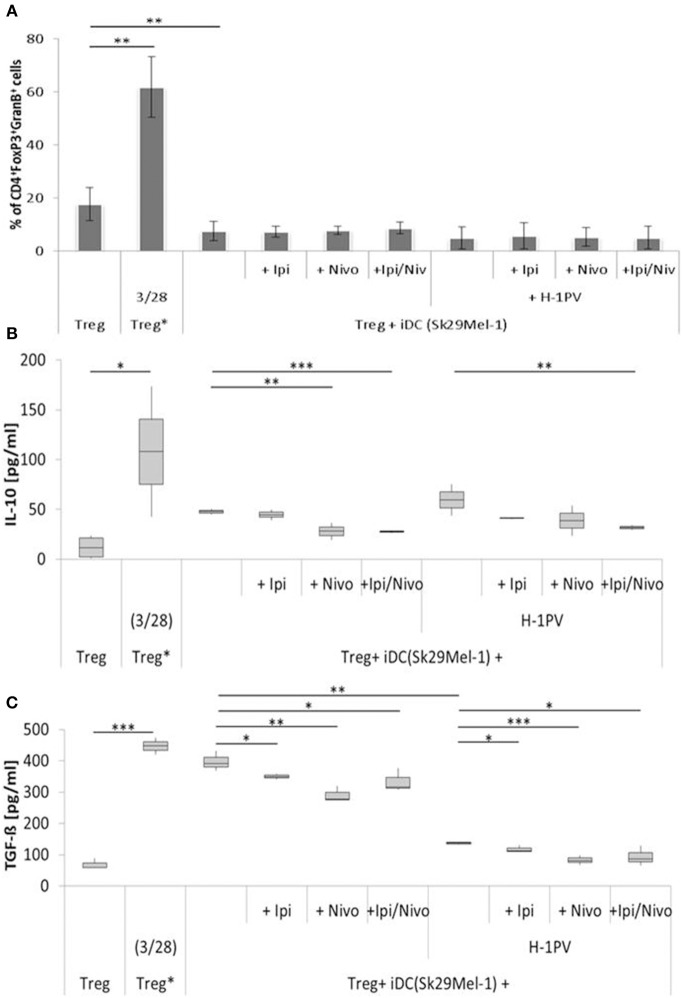
Determination of regulatory T cell (Treg) activity induced by H-1PV infected and uninfected Sk29Mel-1. Treg* functions as positive control and are activated with functional anti CD3 and anti CD28. Measurement of proportion of CD4+FoxP3+GranzymB+ cells by FACS analysis **(A)** and determination of the IL-10 **(B)** and TGF-ß **(C)** level in the supernatant (ELISA) with and without ipilimumab and nivolumab. Experiments were performed independently as triplicate *P ≤ 0.05; **P ≤ 0.01; ***P ≤ 0.001. ELISA, enzyme linked immunosorbent assay; FACS, fluorescence-activated cell sorting.

Tregs are enriched in the TME of melanomas and prevent the activation of T helper cells. The proportion of Tregs induced by H-1PV infected and non-infected Sk29Mel-1 melanoma cells was similar as well as the release of IL-10 (Figure 4). The release of TGF-ß (a measure of Treg activity), was consistently lower when using H-1PV infected melanoma cells (Figure 4).

In co-culture experiments with uninfected Sk29Mel-1 cells, the level of TGF-ß in the supernatant decreased by 12% after adding ipilimumab and by 27% after adding nivolumab and by 16% when ipilimumab and nivolumab were added in combination. Similar effects were achieved on the levels of IL-10. Nivolumab alone and in combination with ipilimumab reduced the amount of IL-10 by 41%. After infection of Sk29Mel-1 with H-1PV, there was no significant change in the level of IL-10 but a decrease in the levels of TGF-ß was detected. In combination with immune checkpoint inhibitors, H-1PV was able to further downregulate the release of TGF-ß and IL-10. While ipilimumab and nivolumab alone caused a small decrease in IL-10, the combination of both inhibitors resulted in a 46% reduction. Comparable results were obtained for TGF-ß. Ipilimumab reduced TGF-ß by 6%, nivolumab by 32% and the combination of both by 31% (Figure 4).

**Figure 5:**
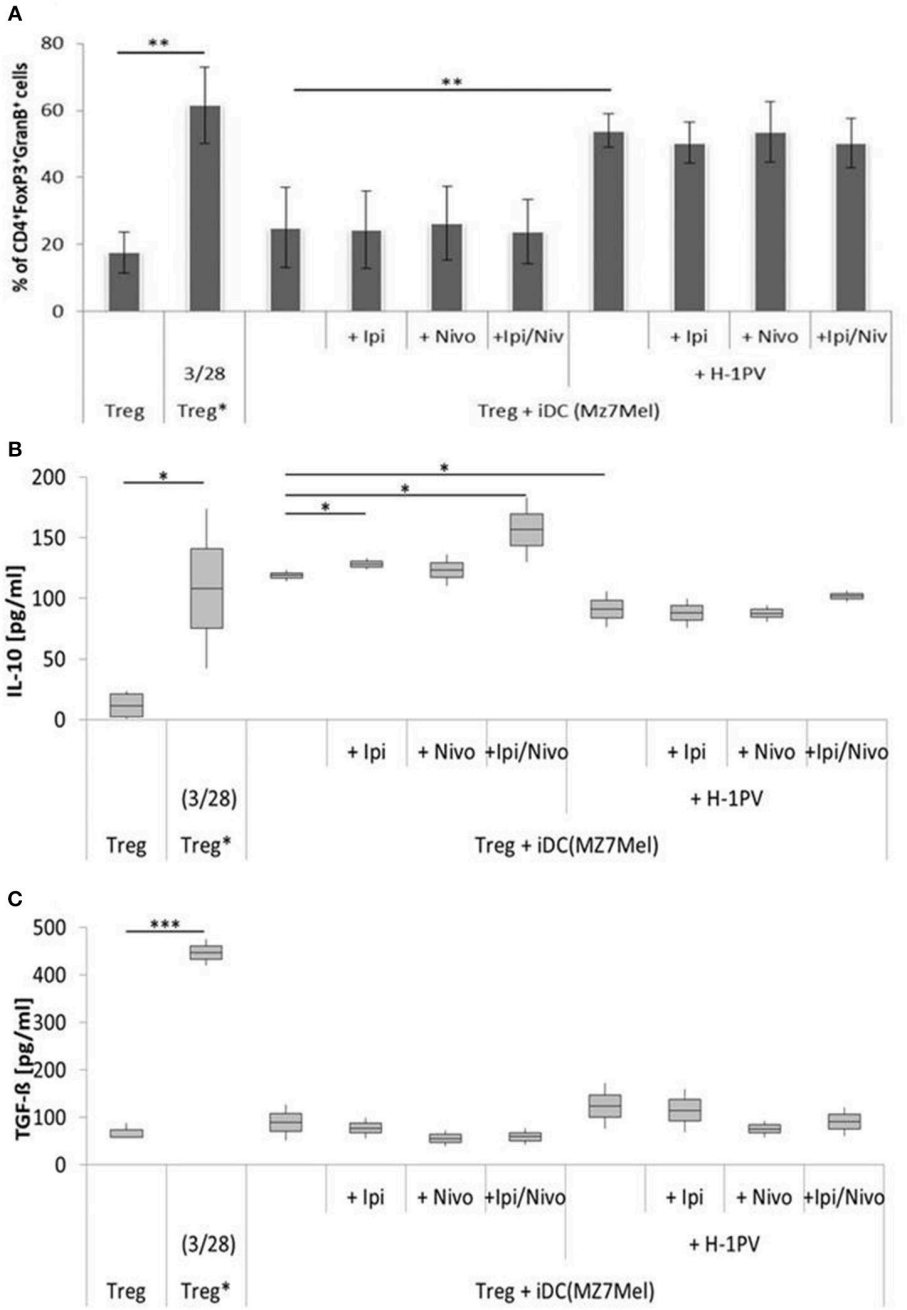
Determination of regulatory T cell (Treg) activity induced by H-1PV infected and uninfected Mz7Mel. Treg* functioned as positive control and are activated with functional anti CD3 and anti CD28. Measurement of proportion of CD4^+^FoxP3^+^GranzymB^+^ cells by FACS analysis **(A)** and determination of the IL-10 **(B)** and TGF-ß **(C)** level in the supernatant (ELISA) with and without ipilimumab and nivolumab. Experiments were performed independently as triplicate *P ≤ 0.05; **P ≤ 0.01; ***P ≤ 0.001. ELISA, enzyme linked immunosorbent assay; FACS, fluorescence-activated cell sorting.

Comparing the two cell lines, co-culture experiments with Mz7Mel demonstrated 2-fold increase in IL-10 and a 5-fold decrease in TGF-ß levels in the supernatant compared with Sk29Mel (Figure 5). The addition of ipilimumab (8%) and ipilimumab with nivolumab (32%) enhanced IL-10 release. The situation was, however, different for TGF-ß. Both checkpoint inhibitors caused a non-significant reduction of TGF-ß in the supernatant. After infection of Mz7Mel with H-1PV, the proportion of Tregs (2-fold) and the level of TGF-ß (27%) increased while the level of IL-10 was 23% lower than in co-cultures with H-1PV uninfected cells. The addition of the immune checkpoint inhibitors did not significantly affect these results. Thus, effects of H-1PV with or without immune checkpoint inhibitors on Treg immune silencing achieved best results in Sk29Mel-1 cells.

### H-1PV Triggered Stronger CTL Activity Combined With Nivolumab or Ipilimumab Compared With Virus Alone

The CTL clone IVSB, which recognizes specific antigens of Sk29Mel-1 and Mz7Mel cells, was used to investigate the effect of H-1PV infection alone and in combination with immune checkpoint inhibitors on CTL activation. As shown in our earlier publications, H-1PV induced high number of lysed cells compared to untreated.

**Figure 6:**
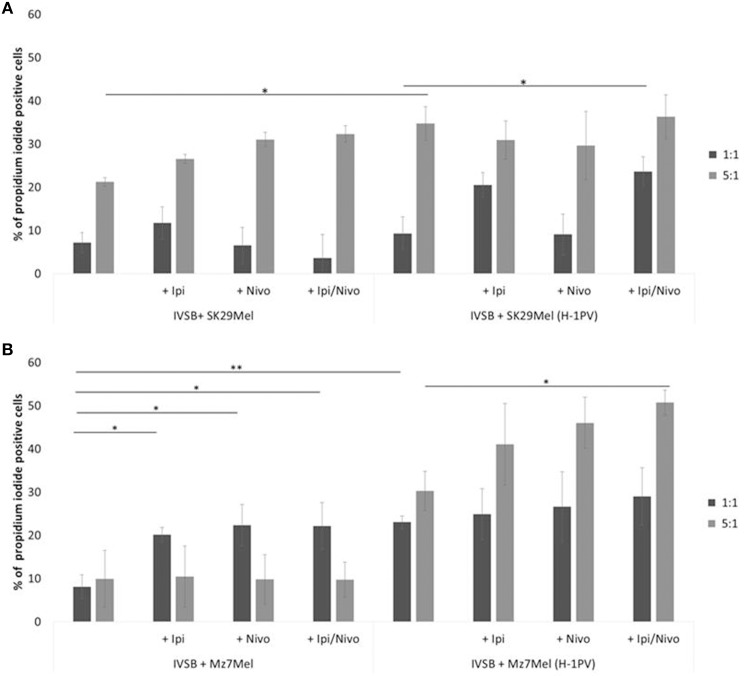
Determination of CTL clone IVSB cytotoxicity induced by H-1PV after 2 days of co-culture with melanoma cells. Measurement of the proportion of CD3^−^CD8^−^CPD^+^PI^+^ cells by FACS analysis. **(A)** Co-culture with Sk29Mel cells and **(B)** with Mz7Mel with and without ipilimumab and nivolumab. Experiments were performed independently three times *P ≤ 0.05; **P ≤ 0.01; *P* ≤ 0.001. CPD, cell proliferation dye eFluor 670; PI, propidium iodide; CTL, human cytotoxic T lymphocytes; FACS, fluorescence-activated cell sorting.

The cytotoxicity of IVSB against both melanoma cell lines benefited from the addition of ipilimumab and nivolumab. There was a trend in increased number of PI^+^ cells, for example, 21% of Sk29Mel were lysed by IVSB in a ratio of 1:5 (Sk29Mel:IVSB; Figure 6A). The addition of ipilimumab (27%) or with nivolumab (32%) was linked to increasing numbers of PI^+^ Sk29Mel. Similar lytic effects were noted with H-1PV infected cells. However, nivolumab alone was not able to reach similar effects.

Analogical findings were measured for Mz7Mel (Figure 6B). IVSB lysed 8% of Mz7Mel and 23% of H-1PV infected Mz7Mel after 72 h in a ratio of 1:1 (Mz7Mel:IVSB). More Mz7Mel cells were lysed when the ratio was enhanced to 1:5 (Mz7Mel:IVSB). Nine percentage of Mz7Mel died without H-1PV and 3-fold more cells were lysed after virus infection. By co-incubation with checkpoint inhibitors, the number of PI^+^ cells tended also to increase (adding ipilimumab, nivolumab, or both together resulted in a 10% increase of PI^+^ cells).

## Discussion

In order to assess the potential in combining oncolytic virotherapy with immune checkpoint inhibition, melanoma cell lines Sk29Mel-1 and Mz7Mel were first analyzed for expression of the immune checkpoint proteins CTLA-4, PD-1, and PD-L1. Both cell lines showed weak expression of all three proteins. However, expression of CTLA-4, PD-L1, and PD-1 was increased in H-1PV-infected melanoma cells. A possible explanation for the increased expression may be the release of checkpoint molecules from intracellular storage locations such as the trans Golgi network, endosomes, secretory granules, and lysosomal vesicles caused by H-1PV infection (37, 38). PD-1 is also stored in intracellular compartments and upregulated on activated T cells. Kleffel et al. showed PD-1 expression in a small subgroup of melanoma cells (39). Through co-expression of PD-1, CTLA-4, and PD-L1, melanoma cells are able to inhibit antigen presenting cells such as DCs by direct binding of CD80 and CD86 through CTLA-4 and by engagement of PD-L1 through PD-1. This may be a cause of the missing recognition and presentation of melanoma-associated antigens toward T helper cells.

Consequently, our analyses investigated the effect of H-1PV infection of melanoma cells on the maturation of DCs. As expected, infection of melanoma cells with H-1PV induced a strong DC maturation response. This effect was not augmented by combination with ipilimumab and nivolumab. Only increased levels of TNFα were detected in combination with ipilimumab in both melanoma cell co-cultures. Expression of maturation markers did not rise correspondingly. In addition to ipilimumab induced increases in TNFα, a nivolumab induced increase in IFNγ was demonstrated in Mz7Mel cells, which was also detectable when ipilimumab was combined with nivolumab. In this case DC maturation was enhanced by the addition of nivolumab and ipilimumab. Both checkpoint inhibitors block the interaction of PD-1 and CTLA-4 on melanoma cells with their counterparts on the surface of DCs. Thus, negative signaling and the induction of DC differentiation toward tolerogenic DCs can be prevented. Tolerogenic DCs impede differentiation of naïve T cells toward Th1 or Th2 and promote Treg development.

Besides tumor cell killing, H-1PV induced maturation of DCs is the first step in the generation of a potent anti-tumor immune response, together with stronger presentation of tumor associated antigens, and was also shown for other oncolytic viruses including vaccinia virus JX-594, measles virus and reovirus (40, 41). This may result in triggering the activation of effector T lymphocytes such as CD4^+^ and CD8^+^ cells. Ipilimumab and nivolumab may play a greater role with regards to generating an efficacious T lymphocyte stimulation, due to the activation induced upregulated surface expression of CTLA-4 and PD-1. Thus, we focused our next analyses on Tregs which are known to express high levels of PD-1 and CTLA-4. In uninfected melanoma cells, increasing numbers of Tregs with corresponding increases in TGF-ß and IL-10 release were detected. The addition of nivolumab in combination with ipilimumab decreased the activity of Tregs in both melanoma cell lines. H-1PV infection of Sk29Mel-1 cells induced a small decrease in the proportion of Tregs and reduced TGF-ß release, which correlates with low activity. The question remains whether ipilimumab and nivolumab in combination with H-1PV induce a benefit in immune stimulation by silencing Treg activity. Our investigations showed a decrease in released TGF-ß and IL-10. H-1PV combined with immune checkpoint inhibitors demonstrated additional silencing of Tregs. The strongest effect was achieved with the combination of ipilimumab and nivolumab on IL10 levels and with nivolumab on TGF-ß in H-1PV-infected Sk29Mel-1 cells. In contrast to these promising data, co-culture experiments with Mz7Mel cells yielded different results. While in Sk29Mel-1 cells IL-10 levels decreased after treatment with ipilimumab and nivolumab, in co-cultures with Mz7Mel levels rose. Infection of Mz7Mel cells increased the number of Tregs which correlated with a lower level of IL10 in the supernatant. Combining H-1PV-infection with checkpoint inhibitors resulted in a tendency for decreased Treg activity. Morales et al. tested the effect of H-1PV on non-activated PBMCs and showed that H-1PV infection does not affect Treg cell survival (11). In summary, H-1PV infection and checkpoint inhibition with ipilimumab and nivolumab may complement each other in silencing Treg induced immune suppression.

We also investigated the effects of H-1PV with and without checkpoint inhibitors on T lymphocyte activation. Morales et al. showed that H-1PV triggers increased activation marker expression (CD69) and cytokine release (IL-2, IFNγ) (11). Similar results were seen in our *ex vivo* melanoma model, Following H-1PV inoculation, number of PI^+^ melanoma cells was increased. Similar results were shown in the clinical trial ParvOryx01 testing H-1PV in glioblastoma (12, 13). Because of the CD8 T cell-derived IFNy release which may drive PD-L1 expression on tumor and immune cells, we had expected a stronger CTL activity after adding nivolumab in our current study (42). In both, the uninfected and H-1PV infected Sk29Mel-1, ipilimumab alone and combined with nivolumab strengthened cytotoxicity. However, nivolumab was not able to reach similar results. Conversely, in co-cultures with the metastatic cell line Mz7Mel cytotoxicity benefited from adding nivolumab. Comparable lytic effects were reached with ipilimumab and both checkpoint inhibitors in Mz7Mel.

In summary, we have shown that H-1PV provides a strong immune stimulating potential. Checkpoint inhibitors strengthened H-1PV and CTL induced tumor cell lysis in these co-cultures. Therefore, the combination of immune checkpoint inhibitors with oncolytic H-1PV may offer a promising approach for the treatment of melanoma patients.

## Author Contributions

KG works in the laboratory of MM, performed the experiments and wrote the manuscript with the support of FF and MM. CD is a member of JR's working group, both are a mandatory part in supporting our work and providing the oncolytic parvovirus H-1.

### Conflict of Interest Statement

The project was partially supported by an educational grant of Bristol-Myers Squibb. JR is co-inventor in patents/patent applications relating to the cooperation between parvoviruses and the immune system. MM has received honoraria from BMS for advisory and lecture commitments. As PI of Phase III studies, he has received travel support from BMS to ESMO or ASCO meetings. The remaining authors declare that the research was conducted in the absence of any commercial or financial relationships that could be construed as a potential conflict of interest.
